# Sparse Gamma Rhythms Arising through Clustering in Adapting Neuronal Networks

**DOI:** 10.1371/journal.pcbi.1002281

**Published:** 2011-11-17

**Authors:** Zachary P. Kilpatrick, Bard Ermentrout

**Affiliations:** Department of Mathematics, University of Pittsburgh, Pittsburgh, Pennsylvania, United States of America; École Normale Supérieure, College de France, CNRS, France

## Abstract

Gamma rhythms (30–100 Hz) are an extensively studied synchronous brain state responsible for a number of sensory, memory, and motor processes. Experimental evidence suggests that fast-spiking interneurons are responsible for carrying the high frequency components of the rhythm, while regular-spiking pyramidal neurons fire sparsely. We propose that a combination of spike frequency adaptation and global inhibition may be responsible for this behavior. Excitatory neurons form several clusters that fire every few cycles of the fast oscillation. This is first shown in a detailed biophysical network model and then analyzed thoroughly in an idealized model. We exploit the fact that the timescale of adaptation is much slower than that of the other variables. Singular perturbation theory is used to derive an approximate periodic solution for a single spiking unit. This is then used to predict the relationship between the number of clusters arising spontaneously in the network as it relates to the adaptation time constant. We compare this to a complementary analysis that employs a weak coupling assumption to predict the first Fourier mode to destabilize from the incoherent state of an associated phase model as the external noise is reduced. Both approaches predict the same scaling of cluster number with respect to the adaptation time constant, which is corroborated in numerical simulations of the full system. Thus, we develop several testable predictions regarding the formation and characteristics of gamma rhythms with sparsely firing excitatory neurons.

## Author Summary

Fast periodic synchronized neural spiking corresponds to a variety of functions in many different areas of the brain. Most theories and experiments suggest inhibitory neurons carry the regular rhythm while being driven by excitatory neurons that spike more sparsely in time. We suggest a simple mechanism for the low firing rate of excitatory cells – spike frequency adaptation. Combining this mechanism with strong global inhibition causes excitatory neurons to group their firing into several clusters and, thus, produce a high frequency global rhythm. We study this phenomenon in both a detailed biophysical and an idealized model that preserves these two basic mechanisms. Using analytical tools from dynamical systems theory, we examine why adaptation causes clustering. In fact, we show the number of clusters relates to a simple function of the adaptation time scale over a broad range of parameters. This allows us to develop several predictions regarding the formation of fast spiking rhythms in the brain.

## Introduction

Synchronous rhythmic spiking is ubiquitous in networks of the brain [1]. Extensive experimental evidence suggests such activity is useful for coordinating spatially disparate locations in sensory [2], motor [3], attentional [4], and memory tasks [5]. In particular, network spiking in the gamma band (30–100 Hz) allows for efficient and flexible routing of neural activity [6]. Groups of neurons responding to a contiguous visual stimulus can synchronize such fast spiking to within milliseconds [7]. The processing of other senses like audition [8] and olfaction [9] has also been shown to employ synchronized gamma rhythms, suggesting this fast synchronous activity is indispensable in solving perceptual binding problems [10]. Aside from sensation, gamma band activity has been implicated in movement preparation in local field potential recordings of macaque motor cortex [3] and electroencephalogram recordings in humans [11]. Also, there is a boost in power of the gamma band in both sensory [12] and motor [13] cortices during an increase in attention to related stimuli, which may serve as a gain control mechanism for downstream processing [4]. Short term memory is another task shown to consistently use gamma rhythms in experiments where humans must recall visual stimuli [14]. Thus, there are a myriad of studies showing gamma band synchrony appears in signals of networks performing neural processing of a variety of tasks and information. This suggests an understanding of the ways in which such rhythms can be generated is incredibly important to understanding the link between single neuron activity and network level cognitive processing.

Many theoretical studies have used models to generate and study fast, synchronous, spiking rhythms in large neuronal networks [15]–[17]. One common paradigm known to generate fast rhythms is a large network of inhibitory neurons with strong global coupling [16]. Periodic, synchronized rhythms are stable because all cells must wait for global inhibition to fade before they may spike again. This observation lends itself to the theory that gamma rhythms can be generated solely by such mutual inhibition, the idea of interneuron network gamma (ING) oscillations [18]. Of course, this idea can be extended to large networks where excitatory neurons strongly drive inhibitory neurons that in turn feedback upon the excitatory population for a similar net effect [19], [20] (see also Fig. 5 of [21]), known as pyramidal–interneuron network gamma (PING) oscillations [18], [22]. Even when coupling is sparse and random, it is possible for large networks with some inhibitory coupling to spontaneously generate a globally synchronous state [19], [23]. The primary role of inhibitory neurons in gamma rhythms has been corroborated *in vivo* by [24], using optogenetic techniques. Light-driven activation of fast-spiking interneurons serves to boost gamma rhythms, whereas driving pyramidal neurons only increases the power of lower frequencies. Depolarization of interneurons by activating channelrhodopsin-2 channels has also been shown to increase gamma power in local field potentials [25]. Still, no conclusive evidence exists to distinguish between PING or ING being more likely, and [26] suggests that weak and aperiodic stimulation of interneurons is the best protocol to make this distinction. Nonetheless, it is clear that recent experiments have verified much of the extensive theory developed regarding the mechanism of gamma rhythms.

One particularly notable experimental observation of the PING mechanism for gamma rhythms is that constituent excitatory neurons fire sparsely and irregularly [12], [27], while inhibitory neurons receive enough excitatory input to fire regularly at each cycle. Due to their possessing slow hyperpolarizing currents, pyramidal neurons spike more slowly than interneurons [28], so this partially explains their sparse participation in a fast rhythm set by the interneurons. Modeling studies have accounted for the wide distribution of pyramidal neuron interspike intervals by presuming sparse random coupling in network connections [29] or by including some additive noise to the input drive of the population [30]. From this standpoint, the excitatory neurons are passive participants in the generation of fast rhythms, so their statistics have no relation cell to cell. The requirement, in these cases, is a high level of variability in the structure and drive to the network. However, an alternative explanation of sparse firing might suggest that excitatory neurons assemble into subpopulations, *clusters*, that fire in a more regular pattern for a transient period of time. This may be accomplished without the need for strong variability hardwired into a network.

One cellular mechanism that has been largely ignored in network models of fast synchronous spiking rhythms is spike frequency adaptation [30], [31]. Slowly activated hyperpolarizing currents known to generate spike frequency adaptation have been shown in many different populations of regular spiking cells within cortical areas where gamma rhythms arise. In particular, pyramidal neurons in visual cortex exhibit slow sodium and calcium activated afterhyperpolarizing current, proposed to play a major role in generating contrast adaptation [32]. Regular spiking cells in rat somatosensory cortex also have adaptive currents. Furthermore, they exhibit a type 1 threshold, where they can fire regularly at very low frequencies [33]. Also, recent experiments in primate dorsolateral prefrontal cortex reveal significant increases in interspike intervals due to spike frequency adaptation [34]. Synchronous spiking in the gamma range has been observed in visual [2], [12], somatosensory [35], [36], and prefrontal [14] cortex, all areas with neurons manifesting adaptation. Also, adaptation may promote a low resonant frequency in regular spiking neurons that participate in gamma rhythms, as revealed by optogenetic experiments [24]. Therefore, adaptation not only slows the spike rate of individual regular spiking neurons, but can play a role in setting the frequency of network level spiking rhythms.

Thus, we propose to study a paradigm for the generation of a network gamma rhythm in which excitatory neurons form clusters. This accounts for the key observation that excitatory cells do not fire on every cycle of the rhythm. The essential ingredients of the network are spike frequency adaptation and global inhibitory coupling. Spike frequency adaptation produces the slow firing of individual cells. The restrictions on the sparsity of coupling and the level of noise in the network are much looser than [30]. After identifying these properties of the network, we can extract several relationships between parameters of our model and attributes of the resulting clustered state of the network. One result of considerable interest is the relationship between the time constant of adaptation and the number of clusters that can arise in the network. Using two different methods of analysis, we can predict the cluster number 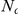 to scale with adaptation time constant 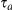 as 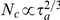.

The paper employs both a detailed biophysical model as well as an idealized model that we study for the formation of cluster states. Our results begin with a display of numerical simulations of cluster states in the detailed model. The main point of interest is that excitatory neurons possess a spike frequency adaptation current whose timescale appears to influence the number of clusters that can arise. To begin to understand how this happens, we analyze the periodic solution of a single adapting neuron, in the limit of large adaptation time constant, for an idealized model of adapting neurons. Using singular perturbation theory, we can derive an approximate formula for the period of a single neuron and thus an estimate of the number of clusters in a network of neurons. Then, an exact expression is derived for the periodic solution of an equivalent quadratic integrate and fire model with adaptation as well as its phase-resetting curve. Next, we employ a weak coupling assumption to predict the number of synchronized clusters that will emerge in the network as the amplitude of additive noise is decreased. The number of clusters in the predicted state is directly related to a Fourier decomposition of the phase-resetting curve. Our main result is that both the singular perturbation theory and weak coupling analysis predict the same 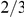 power law relating cluster number to adaptation time constant. Finally, we compare our predictions made using singular perturbation theory and the weak coupling approach to numerical simulations of the idealized model and the detailed biophysical model.

## Methods

### Traub model of an excitatory-inhibitory network with adaptation

For our initial numerical simulations, we use a biophysical model developed by Traub for a network of excitatory and inhibitory spiking neurons [37]. Parameters not listed here are given in figure captions. The membrane potentials of each excitatory neuron and each inhibitory neuron satisfy the dynamics:
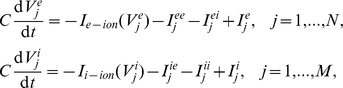
with synaptic currents
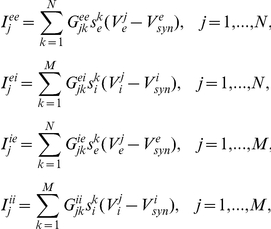
where 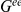, 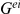, 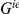, and 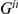 are random binary matrices such that
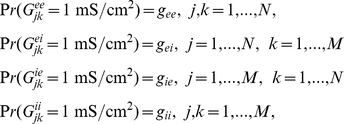
and the synaptic gating variables are given
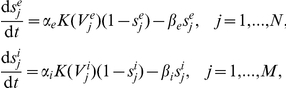
where
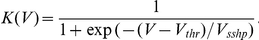
The ionic currents of each excitatory and each inhibitory neuron are given
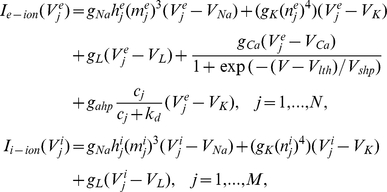
where gating variables evolve as
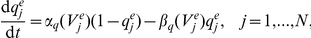


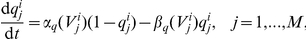
where 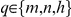. The biophysical functions associated with the gating variables are
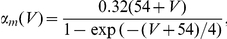


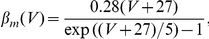


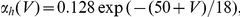


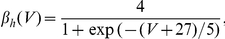


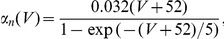


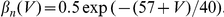
Calcium concentration associated with the hyperpolarizing current responsible for spike frequency adaptation in excitatory neurons follows the dynamics
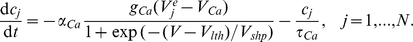
Bias currents to both excitatory and inhibitory neurons have a mean and fluctuating part
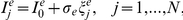


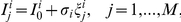
where fluctuations are given by a white noise process such that
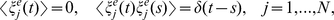


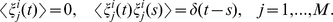
Finally, the fixed parameters associated with the network model are
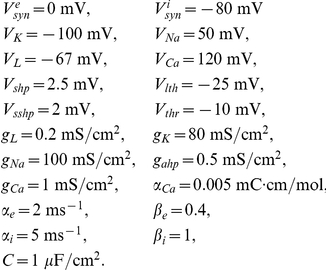
Random initial conditions are used for the simulations of the model, and we wait until the system has settled into a steady state to make calculations of the statistics. We evolve this model numerically, using the Euler-Maruyama method, with a time step of dt = 0.0001.

### Idealized model network with adaptation

The majority of our analysis uses an idealized spiking neuron model to study the mechanism of clustering associated with a network of adapting neurons. The Traub model for a single neuron exhibits a saddle-node on an invariant circle (SNIC) bifurcation. It is possible to exploit this fact to reduce the Traub model to a theta neuron model with adaptation, if the system is close to the bifurcation and the adaptation is small and slow [38]. In [39], an alternative conductance based model with an afterhyperpolarizing (AHP) current was reduced using phase reduction type techniques, where the AHP gating variable was taken to evolve slowly. In particular, Fig. 3(c) of [39] shows that the associated phase-resetting curve has a characteristic skewed shape. We also eliminate the inhibitory cells from the idealization of this section by slaving their synaptic output to the total firing of the excitatory cells. To our knowledge, there is no rigorous *network* level reduction that would allow us to reduce the excitatory-inhibitory conductance based network to the idealized one we present here. We do not provide a meticulous reduction from the Traub network model to the network analyzed from here on. We do wish to preserve the essential aspects of the biophysical model described in the previous section, spike frequency adaptation and inhibitory feedback.

Therefore, we consider a system of 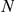 spiking neurons, each with an associated adaptation current, globally coupled by a collective inhibition current
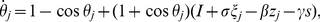
(1a)

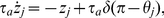
(1b)

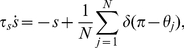
(1c)for 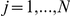. Equation (1a) describes the evolution of a single spiking neuron 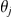 with input 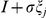, in the presence of spike frequency adaptation with strength 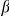 and global inhibition with strength 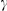. Each neuron's input has the same constant component 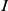 and a unique noisy component with amplitude 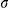 where 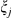 is a white noise process such that 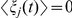 and 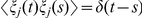 for 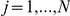. The adaptation current associated with each neuron 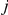 is discretely incremented with each spike and decays with time constant 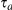, according to equation (1b). Global inhibitory synaptic current is incremented by 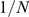 with each spike and decays with time constant 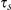. Notice, in the limit of pulsatile synapses (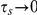), the equation (1c) for inhibitory synaptic current becomes
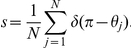
We will make use of this reduction for some calculations relating cluster number to model parameters. The membrane time constant of neurons is usually approximated to be between 1–5 ms, so even though time 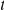 has been nondimensionalized, its units could be deemed to be between 1–5 ms. In addition, experimental results suggest that the hyperpolarizing currents that generate spike frequency adaptation decay with time constants roughly 40–120 ms [40], [41], indicating that 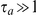. This observation will be particularly helpful in calculating a number of results.

Note, we consider this model as an idealization of adapting excitatory spiking neurons coupled to a smaller population of inhibitory neurons that then collectively connect to the excitatory population. Our approximation is reasonable, considering inhibitory neurons evolve on a faster timescale than the adapting excitatory neurons, as they did in the more detailed biophysical Traub model. For our numerical simulations of this model, we employ the Euler-Maruyama method, with a time-step of dt = 0.0001.

### Calculating spike statistics of the Traub model

**Figure 1:**
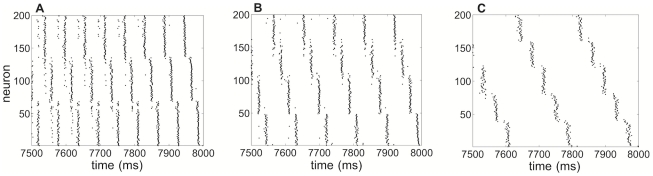
Clustering in numerical simulations of a network of 200 excitatory and 40 inhibitory Traub neurons. Plots of excitatory neuron spike times with neural index sorted according the value of 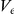 at time 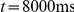. **A** Three clusters providing a total network rhythm of 50 Hz for 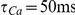, 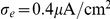, 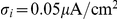. **B** Four clusters providing a total network rhythm of 45 Hz for 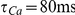, 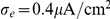, 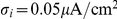. **C** Five clusters providing a total network rhythm of 25 Hz for 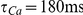, 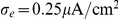, 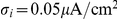. Connectivity parameters are 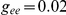, 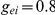, 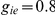, 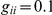 (see Methods for other parameters).

**Figure 2:**
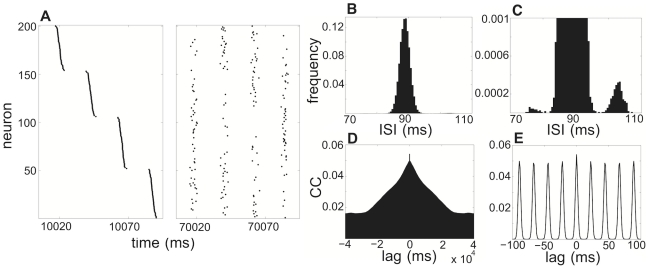
Neurons switch clusters via cycle skipping in a network of 200 excitatory and 40 inhibitory Traub neurons. A network of 200 excitatory and 40 inhibitory neurons with 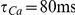 supports four clusters here for a population rhythm of about 45 Hz. **A** Spike times of neurons where index is sorted according to their spike times soonest after 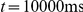. After 60 s, neurons have become thoroughly mixed with other clusters. **B** Histogram showing frequency of interspike interval (ISI) for all excitatory neurons in network. Large peak is just above the calcium time constant 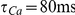, but smaller peak occurs at a higher ISI. **C** By zooming the scale of the frequency into 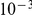, the small peak at larger ISIs is more visible. This suggests neurons switch clusters by skipping one cycle of the fast rhythm. **D** Correlation coefficient plotted over the domain of 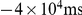 to 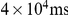 (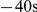 to 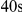). Over long time intervals, correlations degrade, due to noise-induced cycle skipping or (rarer) early spiking. **E** For a tighter lag domain, 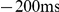 to 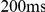 (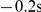 to 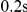), the ringing at discrete lag intervals due to consistent cluster time intervals is apparent. Same parameters are used here as in Fig. 1.

To display the spikes from our simulations of the Traub model (see Figs. 1 and 2), we employ the following sorting technique. First, to better illustrate the formation of clusters, we sort the simulations displayed in Fig. 1 in order of increasing voltage 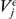 at the end of the simulation using MATLAB's sort function. Similarly, we sort the neurons in Fig. 2a in decreasing order, according to their spike time closest to 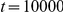 ms also using the sort function. We do not resort the neurons between the left and right panel, which displays the mixing effects of cycle skipping.

We use standard techniques for computing the interspike interval (ISI) and correlation coefficient (CC) for the population of spike trains. Calculations of the ISI take spike times of each neuron 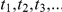 (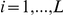) and compute their difference 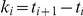 (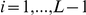). Interspike intervals of all 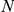 excitatory neurons are then combined into one vector and a histogram is then computed with MATLAB's hist function for a bin width of 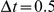. We compute the CC for all possible pairs of excitatory neurons to ensure the best possible convergence. We first digitize two neurons' (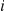 and 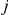) spike trains into bins of 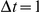 and then use MATLAB's xcorr function to compute an unnormalized correlation function. This is then normalized by dividing by the geometric mean 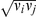 of both neuron's total firing 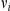 and 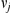 over the time interval. For the calculations displayed in Fig. 2, we use a total run time of 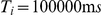.

### Least squares fits to cluster number–adaptation time constant relations

The extensive singular perturbation theory analysis we carry out on the idealized network suggests that there is a clear cut scaling 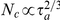 for the relationship between the number of clusters 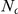 arising in a network and the adaptation time constant 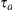 (We also use the following least squares method to fit data relating 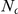 to 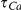 attained from numerical simulations of the Traub model). To compare this result with the relations between 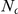 and 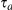 derived using a weak coupling assumption, we consider the function 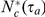 determined by (23). This gives the number of clusters associated with a particular 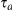 and so must be an integer number. Since (6) is a continuous function, we wish to remove the stepwise nature of 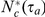 to make a comparison. Thus, we first generate the vector and matrix
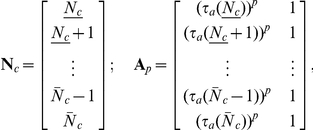
where 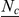 and 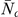 are the minimum and maximum number of clusters attained in the given range of 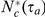. The function 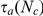 gives the minimal value of 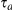 such that 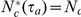; in other words
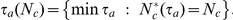
Note that 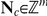 and 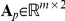. Now, we solve for the coefficients of the power function fit 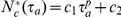 by solving
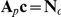
as an overdetermined least squares problem for the coefficient vector 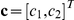. We find the points 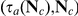 are well fit by the specific case 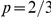. To generate the inset plot, we simply compute the 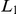 residual
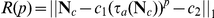
for 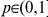. This shows the global minimum is in very close proximity to 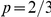.

### Simulating the idealized model

As a means of comparison with our theory, we perform simulations of the idealized model by starting the system (1) at random initial conditions
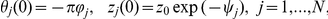
where 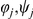 are uniformly distributed random variables on 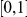, 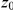 is given in Text S1, and 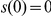. As suggested by our weak coupling analysis, we start the system with high amplitude noise (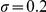), where clusters are not well defined, and incrementally decrease 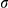 as the system evolves until noise is relatively weak (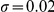). For low noise, each cluster is particularly well defined, especially when there are fewer clusters present.

### Minimal adaptation time constant corresponding to cluster number

We now describe the attainment of the data points corresponding to minimal 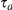 (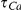 for our calculations of the Traub model) to attain 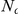 clusters for numerical simulations. These are computed by, first, simulating 20 realizations for each value of 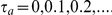 (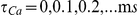 for the Traub model), starting with random initial conditions (2) and high noise, reducing noise and stopping after 20000 time units (20000 ms for the Traub model), and finally recording the number of clusters in the network for each realization. The points we then plot correspond to the first value of 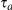 whose median cluster number is larger than the median for the previous 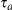 (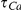) value. Increments in 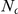 between neighboring 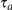 (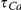) values are always no more than one.

## Results

### Clustering in a network of spiking neurons

Clustering of spiking activity in a network of neurons is the phenomenon in which only neurons belonging to the same cluster spike together, and two or more clusters spike each period of the population oscillation. The emergence of cluster states has been studied in globally coupled networks of phase oscillators with additive noise [42], where clusters can be identified using stability analysis of an associated continuity equation. Phase oscillator networks may also develop clustering in the presence of heterogeneous coupling [43] or time delays [44], [45]. Golomb and Rinzel extended early work in phase oscillators to show cluster states can arise in biologically-inspired networks of Wang–Rinzel spiking neurons [46]. They employed a stability analysis of periodic solutions to their network, using Floquet multipliers to identify which cluster state could arise for a particular set of parameters. Networks of leaky integrate-and-fire neurons can also exhibit clustering if coupled with fast inhibitory synapses [47] or there is sufficient heterogeneity in each neuron's intrinsic frequency [48]. In Hodgkin-Huxley type networks clustering has been witnessed due to a decrease in the amplitude of a delayed rectifier current [16] or by simply including a delay in synaptic coupling [44]. The addition of a voltage dependent potassium current to an excitatory-inhibitory network has also been shown to form two cluster states in detailed simulations [49].

In this section, we show clustering can arise in a detailed biophysical model network of spiking neurons developed by Traub (see Methods). The network consists of excitatory and inhibitory neurons, but only excitatory neurons possess a slow calcium activated hyperpolarizing current, representative of spike frequency adaptation. The connectivity structure is dense but random, where each pair of neurons has a set probability of being connected to one another, according to their type. Here, we present the results of numerical simulations of this model, showing the behavior of cluster states in the network. More specifically, we are interested in the way that spike frequency adaptation helps to generate these states. In later sections, we look at cluster states in an idealized network model in order to analytically study the role of adaptation in the onset of clustering.

We first present spike times of a model network of 200 excitatory and 40 inhibitory Traub neurons in Fig. 1 for two different time constants of the calcium-induced hyperpolarizing current. In particular, we find that, for slower adaptation, there is an increase in the number of clusters, but the overall frequency of the network decreases. This relationship persists over a wide range of model parameters, like network connectivity, synaptic strength, and input to neurons. To aid in the visualization of the clusters, we sort the neurons according to their voltage's value at the end of the simulation (see Methods).

Although the size of the clusters is fairly invariant over time, neurons do not remain in the same clusters indefinitely. In fact, by examining the state of neurons at times significantly before of after the time we sort them according to spike times (see Methods), we find that units of clusters begin to mix with one another, shown in Fig. 2(a). Neurons jump from one cluster to another. The mechanisms by which this can occur are that either a neuron fails to fire with its current cluster and fires with the next cluster or the neuron fires with the previous cluster. This is exemplified by the additional peaks in the interspike interval distribution shown in Fig. 2(c). The correlation coefficient is relatively low on short time scales and decreases significantly over long time scales since neurons skip cycles or spike early due to fluctuations in drive to the network (see Fig. 2(d)). As pictured in Fig. 2(e), on short timescales, excitatory neuron spike times are weakly correlated between clusters, before cycle hopping takes effect. We have found that higher amplitude noise leads to more frequent switching of neurons between clusters. In addition, as the number of clusters increases, each individual cluster appears to be less stable and neurons also hop from one cluster to the next more frequently. We have considered architectures for which the cross correlations between neurons decay more quickly due to sparser connectivity. The main goal of our study, though, is to examine clustering as a complementary mechanism to irregular input and random connectivity for generating sparse firing. This can be contrasted with the degradation of correlations between excitatory neurons on fast timescales in [30], due to strong fluctuations and sparse connectivity in their excitatory-inhibitory network.

Thus, the cluster state that arises in this biophysically based network of spiking neurons appears to be a stable state that exists over a large range of parameters. The essential ingredients are a slow adapting current and inhibitory neurons that only fire when driven by excitatory neurons.

### Analysis of clustering mechanism in an idealized network

The key feature of the detailed biophysical model that makes excitatory neurons susceptible to grouping into clusters is spike frequency adaptation. Few studies have examined the effects of adaptive mechanisms on the dynamics of synchronous states in spiking networks. In a study of two coupled adapting Hodgkin-Huxley neurons, their excitatory synapses transitioned from being desynchronizing to synchronizing as the strength of their spike frequency adaptation was increased [50]. In a related study, spike frequency adaptation was shown to shift the peak of an idealized neuron's phase-resetting curve, creating a nearly stable synchronous solution [51]. The effects of this on network level dynamics were not probed, and, in general, studies of the effects of adaptation on dynamics of large scale neuronal networks are fairly limited. A large excitatory network with adaptation can exhibit synchronized bursting, followed by long periods of quiescence set by the adaptation time constant [52]. Spike adaptation must build up slowly and be strong enough to keep neurons from spiking at all. More aperiodic rhythms were studied in populations of adapting neurons by [53], who showed the population frequency could be predicted by the preferred frequency of a single adapting cell. Adaptation has also been posed as a mechanism for disrupting synchronous rhythms in [54], where increasing the conductance of slow hyperpolarizing currents transitions a network to an asynchronous state. There remain many open questions as to how the strength and timescale of adaptive processes in neurons contribute to synchronous modes at the network level.

We therefore proceed by studying several characteristics of the cluster state as influenced by spike frequency adaptation. First, we study how the period of a single neuron relates to the strength and time scale of adaptation. Then, we find how these parameters bear upon the number of clusters arising in the network of adapting neurons with global inhibition. Approximate relations are derived analytically and then compared to the results of simulations of (1) as well as the Traub model.

### Approximating the periodic solution and cluster number with singular perturbation theory

**Figure 3:**
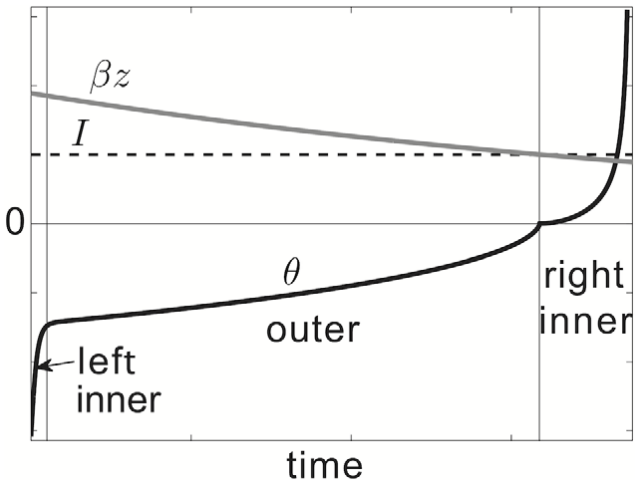
Inner and outer layers of the singular perturbative approximation to periodic solution of idealized model. Singular perturbative approximation to 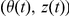, the periodic solution of a single, idealized, spiking neuron model with adaptation (2). See Text S1 for more details on singular perturbative calculation.

We first present a calculation of the approximate period 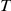 of a single adaptive neuron, uncoupled from the network. The singular perturbation theory we use relies upon the fact that the periodic solution is composed of three different regions in time: an initial inner boundary layer; an intermediate outer layer; and a terminal inner boundary layer. In this case, the initial and terminal boundary layers correspond to what would be the back and front of an action potential in a biophysical model of a spiking neuron, such as the Traub model. The intermediate layer corresponds to a refractory period imposed by the strong slow afterhyperpolarizing current. An asymptotic approximation to the periodic solution is pictured in Fig. 3, showing the fast evolution of 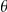 in boundary layers and slow evolution in the outer layer. The slow timescale arises due to the fact that 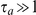, so we shall use the small parameter 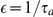 in our perturbation theory. Key to our analysis is the fact that the end of the outer layer comes in the vicinity of a saddle-node bifurcation in the fast subsystem, determined by the 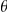 equation (1a). It then turns out that, as a result, we must rescale time to be 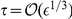 in the terminal boundary solution. Such an approach has been studied extensively by Guckenheimer in the Morris-Lecar and Hodgkin-Huxley neurons with adaptation, as well as general systems that support canards of this type [55], [56]. Nonetheless, we proceed by carrying out a similar calculation here and use it to derive an approximate formula for the period of the solution. We find that it matches the numerically computed solution remarkably well. In addition, we can use the expression for the period to explain why the number of clusters 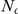 arising in the network (1), when compared to the adaptation time constant 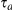, will scale as 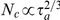.

To initially approximate the interspike interval for a deterministically–driven adaptive neuron, uncoupled from the network
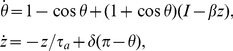
(2)we shall use singular perturbation theory. In particular, we exploit the fact that the adaptation time constant 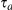 is large in comparison to the membrane time constant of a spiking neuron. Guckenheimer has carried out several other studies examining relaxation oscillations and canards in the vicinity of fold singularities [55], [56]. The usual approach is to decompose the full system into a fast and slow part and then use standard methods of bifurcation analysis to analyze constituent parts [57].

**Figure 4:**
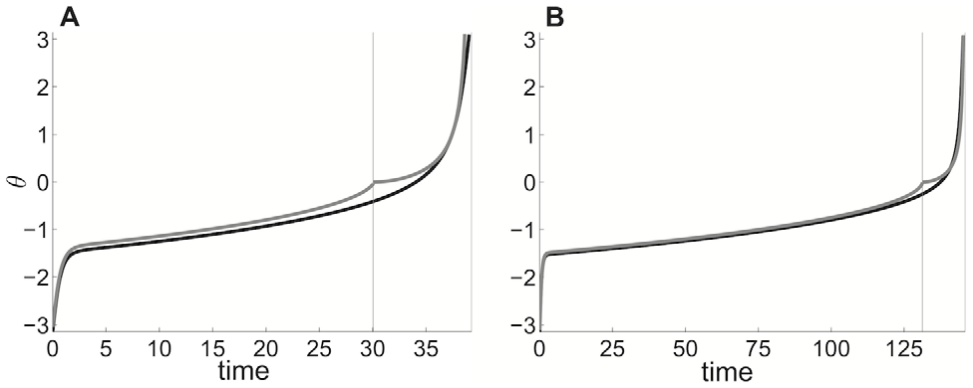
Singular perturbative theory approximates numerically evolved solution of idealized model reasonably well. Comparison of the singularly perturbed solution (grey) and the numerically evolved solution (black) of (2) when the adaptation time constant **A** 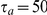 and **B** 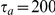. Vertical grey line denotes location of cusp, where a saddle-node bifurcation occurs in fast subsystem at time 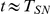 (see Text S1). Other parameters are 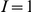 and 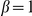.

We are particularly interested in computing the approximate form of a periodic solution. The details of this calculation are carried out in Text S1. Our analysis exploits the fact that the fast subsystem, defined by the 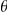 equation of the system (2), exhibits a saddle-node on an invariant cycle (SNIC) bifurcation. Thus, we have an approximate periodic solution that is split into two time regions, one before the subsystem reaches the SNIC at time 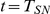 and the other after, so
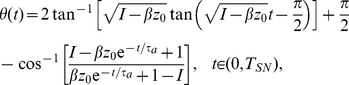
(3)and
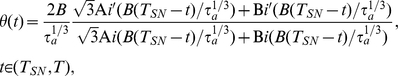
where the parameters 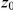 and 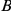 are defined in Text S1 while 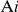 and 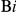 are Airy functions of the first and second kind. We plot this solution along with numerical simulations in Fig. 4. The location of the saddle-node bifurcation point of the fast subsystem correlates biophysically to the end of the refractory period imposed by the afterhyperpolarizing current. Notice that there is a cusp at the point where the outer and terminal boundary solution come together. In addition, the perturbative solution's phase 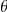 arrives at zero before the actual solution's. This suggests that there are finer scaled dynamics arising from the phase variable being small in the vicinity of the saddle-node bifurcation of the fast subsystem. Such effects could potentially be explored with higher order asymptotics. For the purposes of this study, it suffices to truncate the expansion to two terms. The resulting formulae can be utilized extensively in the explanation of network dynamics.

**Figure 5:**
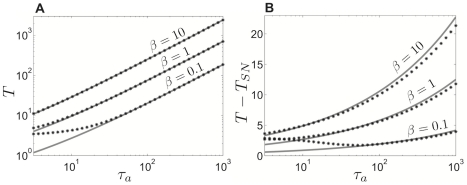
Period of the solution to the single adaptive theta neuron model. We compare the period as calculated by evolving (2) numerically (black stars) against the analytically computed formula for period (4), derived using singular perturbation theory (grey line). **A** Period 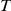 plotted versus adaptation time constant 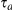. **B** Length of terminal layer 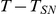 plotted versus adaptation time constant 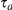. Input parameter 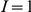.

In deriving our approximation to the periodic solution, we were able to calculate a relatively concise formula relating the period of the solution to the remainder of the parameters
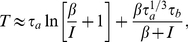
(4)where 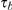 is the minimal solution to
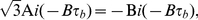
(5)such that 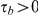 (see Text S1). We illustrate the accuracy of this approximation over a wide range of adaptation time constants 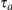 in Fig. 5. The approximation is fairly accurate for a substantial region of parameter space, but improves appreciably as 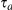 and 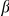 are increased.

We conclude our study of the periodic solution to (2) by using our formula for the period (4) to roughly calculate the number of clusters admitted by a network of adapting neurons with pulsatile inhibitory coupling. This also provides us with an estimate of the population spike frequency. Any inputs delivered to the neuron during the initial or the outer layer stage of the solution, equation (3), will have little or no effect on its firing time. During this interval, the adaptation variable constrains the phase 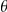 so that it simply relaxes back to the same point on the trajectory following a perturbation. Once the terminal layer begins, the input is above a threshold such that the phase can increase at an accelerating rate. However, it is possible to hold the phase back with a negative perturbation. A neuron that has already begun its terminal phase when another cell spikes will always be forced to delay its own spike. As a result, over time, in a network, clusters of neurons would be forced apart to about the time length of the terminal layer. Therefore, the number of clusters will be roughly determined by the length of this terminal layer as compared with the total length of the period
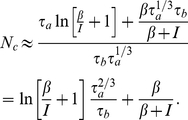
(6)Therefore, as the adaptation time constant increases, the number of clusters will scale as 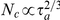. While our main interest in this formula is its relationship to the adaptation time constant, there are also nonlinear relationships derived here between cluster number and other parameters. We shall compare this formula further with the predictions we calculate using weak coupling and the phase-resetting curve. Since the perturbative solution ceases its slow dynamics briefly before the numerical solution (see Fig. 4), we expect that this asymptotic formula (6) approximating cluster size may be a slight underestimate.

**Figure 6:**
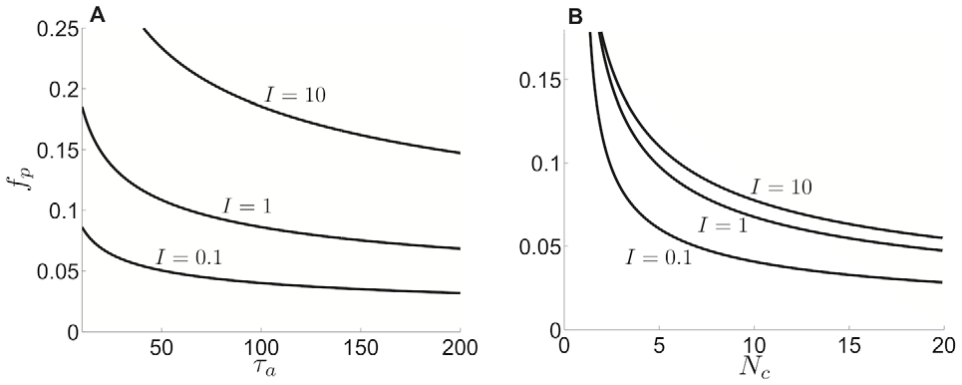
Population frequency's dependence upon cluster number. Plots showing the relationship of the population frequency 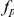 given by equation (7) to **A** the adaptation time constant 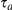 and **B** the cluster number 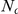 for various values of input 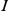. The frequency is given per nondimensional unit of time.

Nonetheless, it allows us to concisely approximate how the population frequency depends on the adaptation time constant 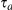 as well as the cluster number 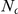. Since each neuron spikes with a period 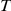 given by equation (4) and there are 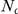 clusters of such neurons, the frequency of populations spikes in the network are given by
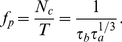
(7)We plot this function versus 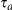 as well as 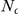 in Fig. 6. Notice, networks with neurons whose spike frequency adaptation have a longer time constant support synchronous spiking rhythms with lower frequencies, as in the Traub network (see Fig. 1). Also, by our mechanism, as more clusters are added, the population frequency decreases. This is due to the period of individual neuron spiking scaling more steeply with adaptation time constant than the cluster number.

We have identified general relationships between the adaptation time constant and two quantities of the idealized spiking network (1): the period of a single neuron and the cluster number of the network. These relationships help characterize the behavior of the cluster state in the adaptive network. In particular, the bifurcation structure of the fast-slow formulation of the single neuron system guides the identification of a 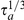 timescale of the spike phase, which evidently guides network level dynamics. Singular perturbation theory is indispensable in making this observation.

### Phase-resetting curve of an adapting neuron

As a means of studying the susceptibility of a single neuron to synchronizing to input from the network, we shall derive the phase-resetting curve of a neuron with adaptation. Biophysically, the phase-resetting curve corresponds to the amount that brief inputs to a tonically spiking neuron delay or advance the time of the next spike. First, we make a change of variables 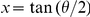 to the system (2), so the state of the neuron is now described by the quadratic integrate and fire (QIF) model with adaptation [58]

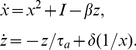
(8)We show in Text S1 that by using a sequence of further changes of variables, we are able to express the periodic solution to this system in terms of special functions. As has been shown previously, the solution to the adjoint equations of a system that supports a limit cycle is the infinitesimal phase-resetting curve (PRC) of the periodic orbit [59]. Therefore, with the function form of 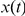 in hand, we can derive the adjoint equations by first linearizing the system (8) about the limit cycle solution 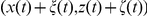 so
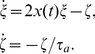
(9)The adjoint equations, under the inner product
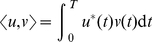
(10)will be
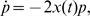
(11)

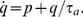
(12)Since 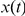 is known, it is straightforward to integrate (11), to solve for the first term of the adjoint
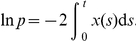
By plugging in 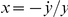 (see Text S1), we find we can further specify
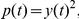
where 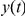 is given up to a scaling factor in Text S1.

**Figure 7:**
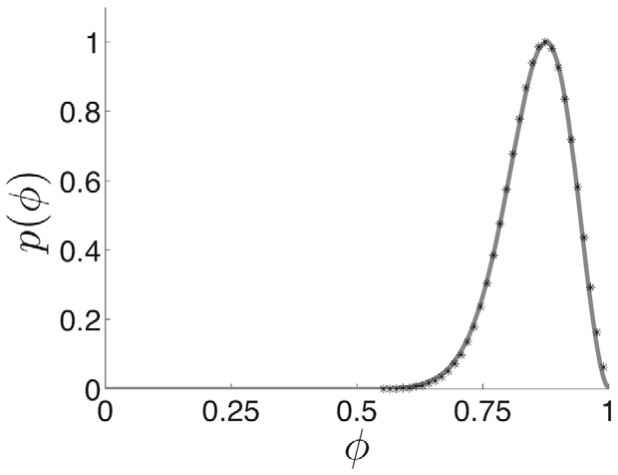
Exact phase resetting curve of quadratic integrate-and-fire model with adaptation. Phase-resetting curve of the QIF model calculated exactly by solving (8) for the periodic solution (grey line) and numerically by applying pulsatile inputs to numerical simulations of (8). Parameters are 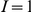, 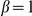, and 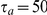.

It is now straightforward to plot the PRC of the QIF model with adaptation. To our knowledge, this is the first exposition of an analytic calculation of the PRC of the QIF model with adaptation. Although, the bifurcation structure of more general QIF models with adaptation has been analyzed in previous work by [60], [61]. The exact period 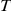 can be computed using the right boundary condition given in Text S1, which can then be used to determine the initial condition for the adaptation variable
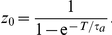
We then must plot a function which involves a Bessel function of imaginary order and imaginary argument
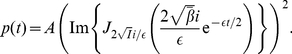
(13)In Fig. 7, this is shown along with the numerically computed PRC, where pulsatile inputs are applied at discrete points in a simulation. Time is also normalized by the period 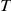 to yield the phase variable 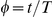. We find an excellent match between the two methods.

**Figure 8:**
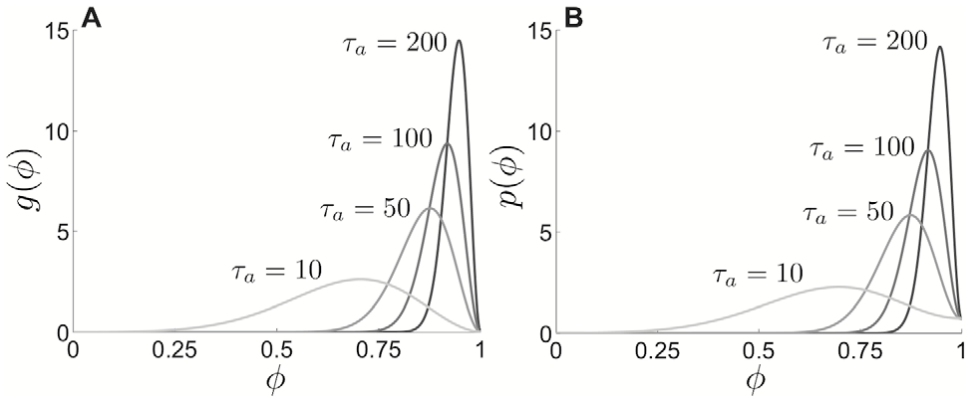
PRCs calculated numerically using the adjoint equation. PRCs calculated for the **A** QIF model with adaptation and **B** theta model with adaptation as function of phase for various adaptation time constant 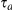 values. For visualization purposes, the PRCs have all been normalized to integrate to unity. Other parameters are 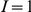 and 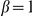.

One can also derive a very accurate representation of the PRC by numerically solving the adjoint equations (11) and (12). This is also useful because Bessel functions with pure imaginary order and argument are particularly difficult to approximate as the magnitude of the order and argument become large. Accurate asymptotic approximations for this class of special functions are lacking, although [62] provides some useful formulae along these lines. Thus, we compute the PRC using numerical solution of the QIF system (8) and the adjoint equation (11), pictured in Fig. 8 for several different 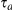 values. Time is normalized here, as in Fig. 7, so the phase variable 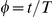 goes between zero and one. This also eases comparison for different time constants 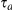. We find that, as we would suspect from our singular perturbation theory calculations, the region in which the neuron is susceptible to inputs shrinks as 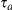 increases. This skewed shape to the PRC has been revealed previously in other studies of spiking models, where adaptation currents were treated in alternative ways [51], [63]. We also compute the PRC for the theta model numerically using the adjoint equations. To derive them, we linearize the system (2) about the limit cycle solution 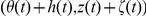 so
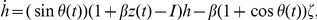


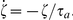
The adjoint equations, under the inner product (10) will be
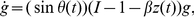
(14)

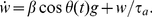
(15)By solving (2) numerically, we can use the solution to then numerically integrate (14) to solve for the first term of the adjoint 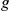, which is the PRC of the theta model. We show this alongside the numerically calculated PRCs of the QIF model. Notice they are quite alike, save for the theta model's PRC being nonzero at 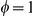. In the theta model's PRC, the change of variables 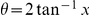 creates a discontinuity.

Therefore, as revealed by an analytic formula and numerical method for computing the PRC, we find that spike frequency adaptation creates a lengthy time window during which the neuron is insensitive to inputs. As the time constant of adaptation 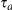 is increased, this window occupies more of the solution period. With these formulations of the PRC in hand, we may carry out a weak coupling analysis of the network to quantitatively study predictions regarding solutions that emerge from instabilities of the incoherent state.

### Weak coupling theory predicts cluster number

Due to large scale spiking network models usually being analytically intractable, a weak coupling assumption is commonly used to study their resulting activity patterns. This allows the reduction of each cell's set of equations to a single one for the phase [38]. Based on the averaging theorem, this reduction is valid as long as parameters of the model are such that each unit supports a limit cycle, their firing rates are not too heterogeneous, and coupling between units is not too strong [15], [59]. This also allows us to place our work in the context of previous studies of clustering in phase models [42]–[44].

Presuming the cells receive enough input to spontaneously oscillate and that they are weakly coupled, we can reduce the system to a collection of limit cycle oscillators [38]. Each oscillator will have some constant frequency 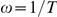, where we use the period computed using the exact solution (see Text S1) for a particular set of parameters. Thus, the network becomes
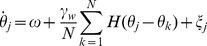
(16)where 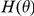 is the coupling function attained by convolving the PRC with the synaptic timecourse
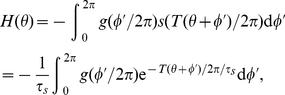
(17)and 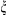 is a white noise process such that 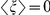 and 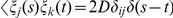. To analyze the system (16), we consider the mean field limit 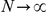. Mean field theory has been used extensively to study (16) when 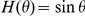 [64]–[66], but much less so when 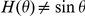 [67], [68]. Following such previous studies, we can employ a population density approach where oscillators are distributed in a continuum of phases 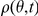 so that 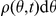 denotes the fraction of oscillators between 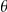 and 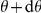 at time 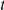. Thus, 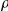 is nonnegative, 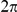-periodic in 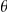, and normalized
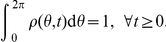
Therefore, 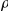 evolves according to the Fokker-Planck equation [64], [65]

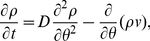
(18)where the instantaneous velocity 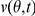 of an oscillator is
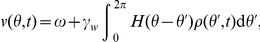
the continuum limit of 
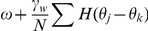
.

**Figure 9:**
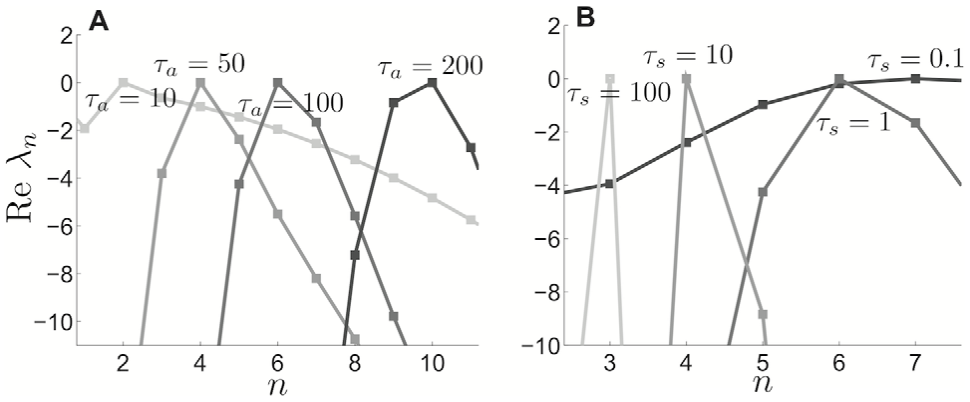
Eigenvalues associated with linear stability of incoherent state predict cluster number. Plots show real part of eigenvalues Re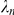 at the critical noise amplitude 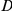 at which the incoherent state destabilizes. **A** When the adaptation time constant is varied as 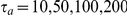, the corresponding predicted number of clusters, in the weak coupling limit, is 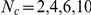 respectively given by (23). Synaptic time constant 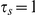. **B** When the synaptic time constant is varied as 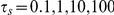, the corresponding predicted number of clusters, in the weak coupling limit, is 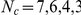. Adaptation time constant 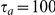. Other parameters are 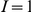, 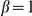, and 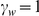.

**Figure 10:**
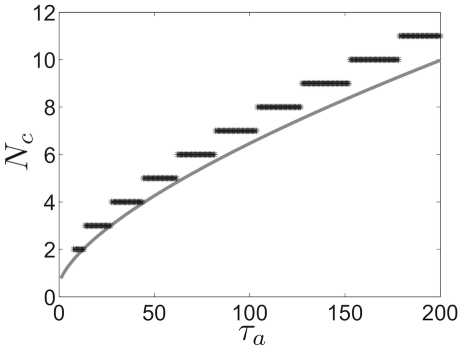
Weak coupling and singular perturbation approximations of cluster number. Cluster number 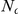 approximations comparison between that given by weak coupling (black stars) – equation (23) – and that given by singular perturbative approximation (grey line) – equation (6). For purposes of comparison, we use pulsatile coupling (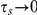) for weak coupling approximation. Other parameters are 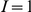 and 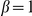.

**Figure 11:**
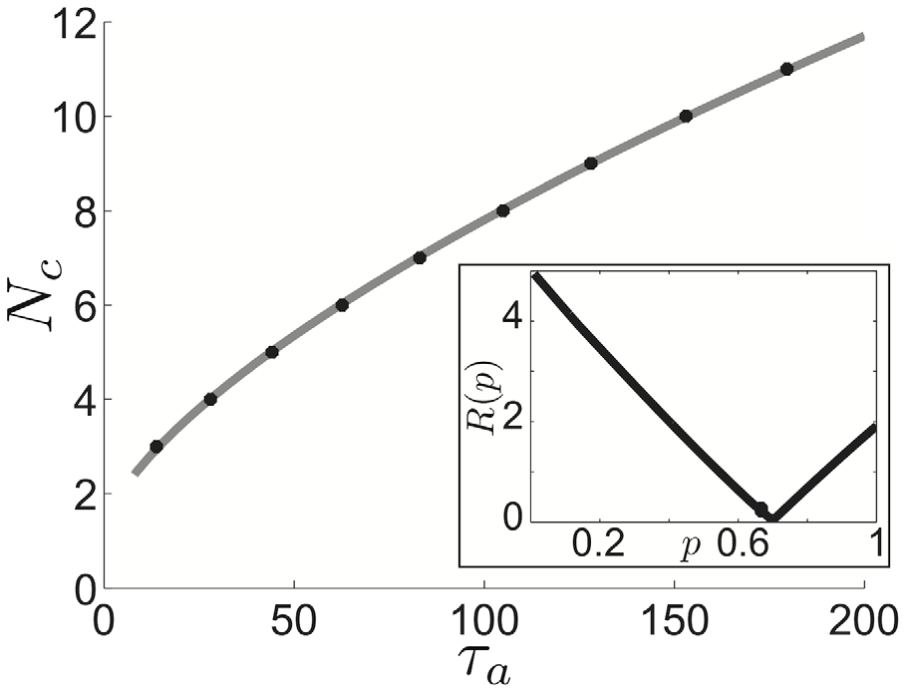
Cluster number computed using weak coupling scales as 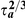
 **.** Cluster number 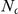 computed using weak coupling formula (23) scales as 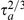 power law for pulsatile coupling (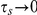). Points 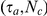 having minimal adaptation time constant 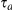 predicting the given cluster number 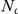 (black stars) calculated with equation (23). A power law function 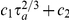 (grey line) is fit to these points using a least squares method (see Methods). (Inset) The 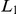 residual 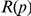 of least squares fits of the points 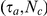 using the function 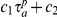 plotted for 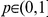 (see Methods). Notice the minimum of 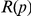 is very close to 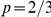.

**Figure 12:**
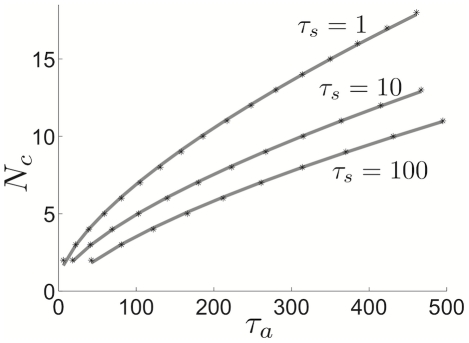
Dependence of cluster number on synaptic time constant. Cluster number 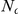 computed as a function of 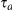 for various synaptic time constants 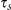 using (23) the weak coupling formula (black stars) and fit to formula 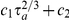 (grey lines) using least square method (see Methods).

Now, in order to examine the effect that the phase-resetting curve has upon the solutions to (16), the weak coupling approximation to (1), we shall study instabilities of the uniform incoherent state of (18), given by 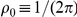. It is straightforward to check that this is indeed a solution by plugging it into (18). Since this is always a solution, for all parameters, we can examine the solutions that emerge when it destabilizes by studying its linear stability. We will show that for 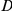 sufficiently large, the incoherent state is stable, but as 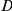 is reduced, the solution destabilizes, usually at a unique Fourier eigenmode. We begin by letting
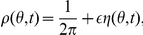
where 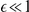. Expanding the continuity equation (18) to first order in 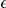, we arrive at an equation for the linear stability of the incoherent state
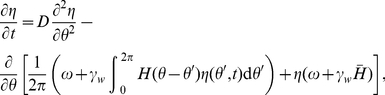
(19)where 
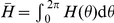
. Expressing 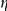 as a Fourier series
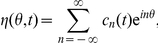
and specifically taking 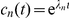, we can compute the eigenvalue 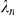 of the 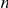
th mode of 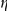 using the spectral equation of the linear system (19), so
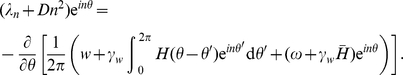
Applying the change of coordinates 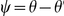, we have a general equation for the 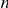
th eigenvalue
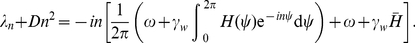
(20)We can evaluate the integral term by considering the Fourier series expansion
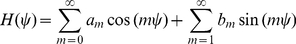
(21)so that
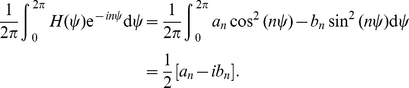
Upon plugging this into (20), we find the eigenvalue associated with the 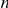
th mode of 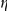 is related to the Fourier coefficients 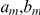 of 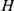 by
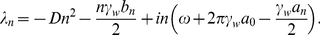
(22)Thus, as 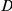 is reduced towards zero, the first eigenmode to destabilize will be the one whose eigenvalue crosses from the left to the right half of the complex plane first. Using equation (22), we can identify this mode as the first 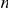 to have Re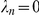 or
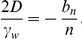
This corresponds to the 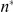 for which 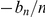 is maximal. For the critical 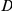 value at which the first eigenvalue has positive real part, we show plots of 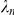 as a function of 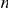 for several different parameters in Fig. 9. Notice that as the adaptation time constant 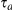 is increased, and other parameters are held fixed, the critical 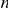 increases. As the synaptic time constant 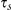 is increased and other parameters are held fixed, the critical 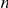 decreases. We contrast this with the case of excitatory coupling (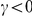) in the system (1), where the PRC is nonnegative. In this case, the critical 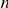 is fairly insensitive to changes in the time constants, virtually always predicting the 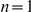 mode becomes unstable first (not shown). Therefore, our weak coupling calculation approximates the number of clusters 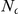 for a given set of parameters using the coupling function (17) with the Fourier expansion (21) so that
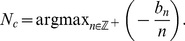
(23)To compare with our singular perturbation theory results, we compute the approximate number of clusters using the weak coupling assumption for pulsatile synapses. In the limit 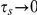, the coupling function becomes 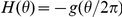. Therefore, the Fourier coefficients 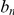 are calculated directly from the PRC of the theta model. In Fig. 10, we plot the number of clusters 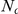 as a function of 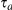, calculated using equation (23) along with the asymptotic approximation to the number of clusters (see equation (6)). Notice that the singular perturbation theory slightly underestimates 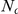 as compared with weak coupling. This may be due to the fact that the singular perturbative solution reaches the saddle-node point slightly before the actual solution does, underestimating the length of the quiescent phase of the PRC. Nonetheless, both curves have a characteristic sublinear shape. We show in Fig. 11 that the weak coupling 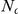 dependence upon 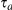 scales as a 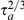 power law, just as predicted by singular perturbative theory. Thus, even though our asymptotic approximation (6) is an underestimate, it provides us with the correct scaling for cluster number dependence upon adaptation time constant. The same power law scaling is reflected in networks with exponentially decaying synapses, as shown in Fig. 12. We plot predictions based on our weak coupling assumption for 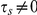. As the synaptic time constant is increased, the number of clusters is diminished, since feedback inhibitory inputs relax more slowly. Therefore, we speculate an improved asymptotic approximation of cluster number that accounts for synaptic timescale might include an inverse dependence upon 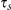.

### Comparing numerical simulations to theoretical predictions of clustering

In this section, we present results of numerical simulations of the idealized network (1) of theta neurons with global inhibition and adaptation. In addition, we compare the scaling law predicted for the idealized model to the number of clusters arising in numerical simulations of the more detailed Traub model. We find that the qualitative predictions of our singular perturbation theory and weak coupling approximations are reflected in the dependence of the state of the network on model parameters. The quantitative relationship between adaptation time constant and cluster number is sensitive to the strength of global inhibitory feedback 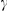, holding for small values only. One would expect this, since approximations were made considering weak coupling.

**Figure 13:**
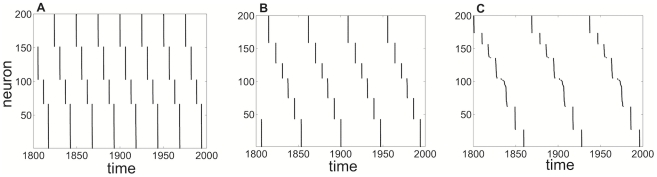
Numerical simulations of idealized network with pulsatile coupling reveal clustering. Cluster states in idealized network (1) with pulsatile coupling (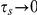). The number of clusters increases sublinearly with adaptation time constant: **A** 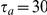, four clusters; **B** 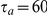, six clusters, **C** 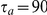, seven clusters. Other parameters are 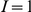, 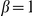, 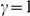, 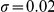.

**Figure 14:**
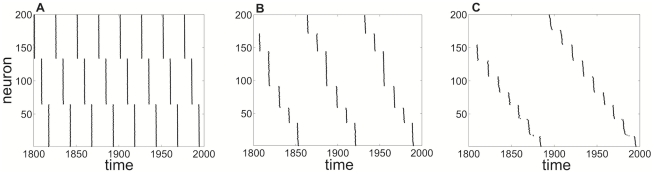
Numerical simulations of idealized network with exponentially decaying synapses reveal clustering. Cluster states in idealized network (1) with exponentially decaying synapses with time constant 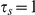. Increasing 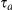 leads to fewer clusters than in the pulsatile synapse case: **A** 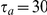, three clusters; **B** 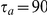, six clusters; **C** 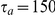, nine clusters. Other parameters are 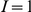, 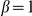, 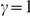, 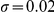.

In Fig. 13, we show the results of simulations for various adaptation time constants in the case of pulsatile synapses (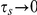). As predicted by the formulae of both our singular perturbation theory approximation (6) and weak coupling assumption (23), cluster number increases sublinearly with adaptation time constant. Notice in Fig. 13(c), when there are seven clusters, neurons of each cluster do not spike in as tight of a formation as can be found in simulations with four and six clusters. We conjecture that this is due to fewer neurons participating in each cluster and so less global inhibition is recruited each time a set of neurons fires. This smears the boundary between each cluster. In Fig. 14, we show the results of simulations in the case of exponentially decaying synapses with time constant 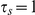. As predicted by our weak coupling analysis, the smoothing of the synaptic signal leads to there being fewer clusters on average for a particular 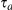 value. Notice in both the pulsatile and exponential synapse cases, as the number of clusters increases, the interspike intervals are prolonged, as predicted by our approximation of the period (4). Therefore, the resulting frequency of population activity decreases, on average, with 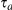.

**Figure 15:**
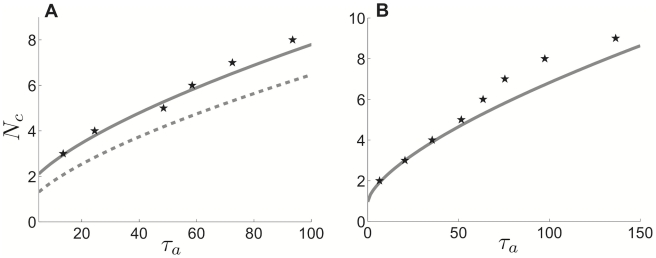
Comparison of cluster number relationship to adaptation time constant in theory and numerical simulations in idealized model. Minimal 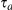 value at which 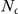 clusters appear in network, a comparison of theory and numerical simulations. Solid grey lines denote theory predicted by weak coupling analysis (fit using least squares approach in Methods). **A** Pulsatile synapses, as predicted by singular perturbation theory (dashed grey) and weak coupling (solid grey); compared with numerical simulations (black stars). **B** Exponential synapses with 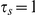. Other parameters are 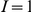, 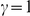, 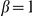.

To quantitatively compare our theoretical predictions with numerical simulations of (1), we plot the minimal 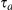 necessary to generate the number of clusters 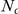 for each method. Theoretical calculations include both the singular perturbation approach (6) and the weak coupling approximation (23). The points we then plot in Fig. 15 correspond to the first value of 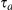 whose median cluster number is larger than the median for the previous 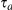 value (see Methods). Remarkably, the theoretical calculation using the weak coupling approach give a reasonable approximation to the behavior of the simulations. Comparing the result of pulsatile versus exponentially decaying synapses, the increase in 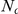 with 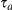 is clearly larger for the pulsatile synapse case. This can be contrasted with the results of van Vreeswijk, who found in simulations of inhibitory integrate and fire networks that median cluster number increased with synaptic timescale [47]. One particular aspect of simulations of the full model (1) that may escape our theoretical formulae (6) and (23) is the effect of different synaptic strengths. To produce fairly well resolved clusters, it was necessary to take 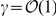, not very weak. Additionally, as the number of clusters increases, the strength of inhibitory impulses decreases. Both of these facts may bear upon potential cluster number and account for the nonlinear shape of the numerically developed relationship between 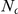 and 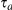.

**Figure 16:**
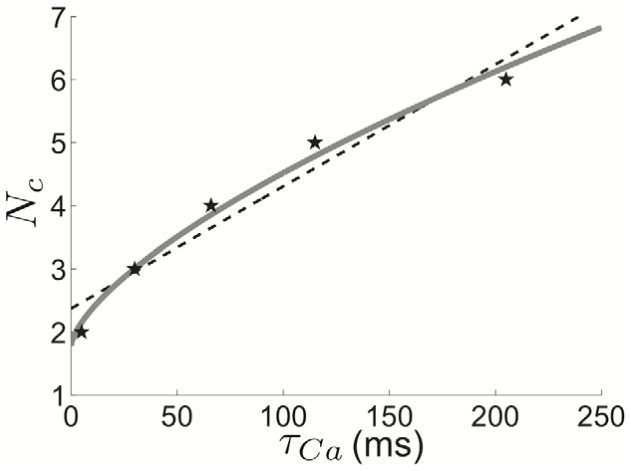
Comparison of cluster number relationship to calcium time constant in theory and numerical simulations in Traub model. Minimal 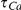 value at which 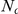 clusters appear in the Traub network. We fit data gathered from numerical simulations (black stars) to two different power functions using a least square method (see Methods). Notice, the least squares fit to 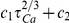 (grey solid) is much better than the fit to a linear function 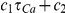 (dashed black). Predictions of cluster scaling (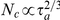) derived for our idealized model (1) carry over with impressive accuracy to simulations of a detailed biophysical model. Connectivity parameters are 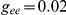, 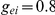, 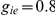, 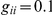 (see Methods for other parameters).

Finally, we return to the original detailed biophysical model to compare the predictions of cluster scaling made in the idealized model. Exchanging the idealized adaptation time constant 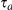 for the time constant for calcium dynamics in the Traub model, 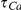, we examine how well the scaling 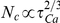 holds in numerical simulations of the detailed model. We use the same method as that employed for the idealized model to identify the minimal 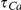 at which a certain number of clusters appears (see Methods). Our results are summarized in Fig. 16 and show that, in fact, cluster number does approximately follow the adaptation time constant scaling predicted from the idealized model. This makes sense, since one can relate the Traub model to the idealized theta model using a normal form reduction, so their phase-resetting properties will be similar to a first approximation [38]. The quiescence invoked by strong adaptation will lead to sharp narrow peaks in the PRC for the Traub model (as shown for the idealized model in Fig. 8(b)). Therefore, our analysis of the theta model leads to an excellent prediction of the effects of adaptation upon the cluster state in the network of Traub neurons.

## Discussion

In this paper, we have studied the formation of cluster states in spiking network models with adaptation. We theorize clustering may be an alternative, or at least contributing, mechanism for the sparse firing of pyramidal cells during gamma rhythms [12]. Sparse gamma rhythms may, therefore, not rely solely upon the effects of input and connectivity heterogeneities [30]. Besides spike frequency adaptation, the other essential property for the formation of clusters in the network is feedback inhibition. Empirically, we observe the number of clusters increases with the time constant of adaptation in a detailed biophysical spiking network and a more idealized model. We can carry out a number of analytical calculations on the idealized model that help uncover the mechanisms of clustering. Results of a singular perturbative approximation of a single neuron's periodic spiking solution confirm that adaptation with longer timescales will shorten the relative length of time a neuron is susceptible to inputs. This is revealed in a compact expression (4) relating the period of the neuron to parameters. In particular, we can estimate the number of clusters 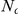 generated in the network for a particular value of adaptation time constant 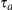 and find they will scale as 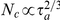. We then compare this result to a formula that can be derived in the context of a phase model, where, incidentally, the phase-resetting curve can be computed exactly. In the weak coupling limit, the number of clusters is related to the Fourier modes of the phase-resetting curve. In fact, we can fit the number of clusters to a 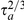 power law. These results are confirmed in simulations of the full idealized model (1) and are well matched to simulations of the detailed biophysical model.

Our results suggest a number of experimentally testable predictions. We have suggested that clustered states may be an organized synchronous state capable of generating sparse gamma rhythms [1]. Rather than a rhythm generated by a balanced network containing neurons with driven by high amplitude noise [30], gamma may be a rhythm generated by slow excitatory neurons that cluster into related groups temporarily but dissociate from one another after some length of time. This could be probed using multiunit recordings to look for clustering of pyramidal neurons on short timescales. Large networks that exhibit clustering may do so through this combination of adaptation and inhibition. This suggests that it may be possible to identify *in vitro* or *in vivo* clustering that depends upon spike frequency adaptation by examining the effects of curtailing calcium dependent potassium currents using cadmium, for example [69]. Our model suggests weakening spike frequency adaptation should lead to a decrease in cluster number. In addition, there are a growing number of ways to experimentally measure the PRC of single neurons [63], [70]. Since pyramidal cells are known to often possess adaptation currents, it may be possible to study the ways in which modulation of those currents' effects bears on a neuron's associated PRC. Our analysis indicates that stronger and slower spike frequency adaptation leads to PRCs with a steep peak at the end. Thus, different aspects of the cluster state shown here may be studied experimentally in several ways.

Clustering through intrinsic mechanisms may in fact be a way for networks to generate *cell assemblies* spontaneously [71]. If clustering is involved in the processing of inputs, shifting neurons from one cluster to another might disrupt the conveyance of some memory or sensation [10], [14]. In more specific networks, underlying heterogeneous network architecture may provide an additional bias for certain neurons to fire together. Alternatively, cell assemblies may be formed due to bias in the input strength to a recurrent excitatory-inhibitory network, as shown in [49]. They found that the inclusion of hyperpolarizing current could generate slow rhythms in the excitatory neurons with increased input. Our model does rely on a hyperpolarizing current but does not require a heterogeneity in the input. Also, each assembly possesses its own beta rhythm whereas the entire network possesses a gamma rhythm.

In the future, it would be interesting to pursue a variety of the theoretical directions suggested by our results. The singular perturbation calculation follows along the lines of a few previous studies of canards in the vicinity of fold singularities [55]–[57], [72]. Carrying out an even more detailed study of the bifurcation structure of the fast-slow system of the single neuron (2) may allow for a more exact calculation of how the period relates to the parameters. In particular, we may be able to compute the dynamics of relaxation time in the vicinity of the bottleneck near the saddle-node bifurcation of the fast system (see Fig. 3). We could also extend this calculation to other idealized spiking models with adaptation such as Morris-Lecar [55] or the quartic integrate and fire model [60]. In addition, we have considered examining the types of dynamics that may result in inhibitory leaky integrate and fire networks with adaptation. Excitatory integrate and fire networks have previously been shown to support synchronized bursting when possessing strong and slow enough adaptation [52]. It has also been shown that inhibitory integrate and fire networks without adaptation support clustering in the case of alpha function synapses [47]. In preliminary calculations, we find that a single integrate and fire neuron with strong and slow adaptation does not have the same steep peaked PRC as the theta model, due to there being no spike signature in the model. Therefore, it may not support clustered states through the same mechanism as the system we have studied. We have also mentioned that clustering arises in the network (1) through the application of a homogenous deterministic current with some additive noise. Therefore, applying an input with more temporal structure, for example at the frequency of the network or individual neurons, may lead to interesting variations of the clustered state. Finally, we seek to study other potential negative feedback mechanisms for generating clusters. In a large competitive spiking network, it may be possible for a subset of neurons to suppress the rest until synaptic depression exhausts inhibition. Multistable states supported with such mechanisms have been shown in small spiking networks [73], [74], but theory has yet to be extended to large scale synchronous states like clustering.

## Supporting Information

Text S1 Singular perturbation theory and exact calculation of periodic solution to idealized spiking model with adaptation.
(PDF)
