# Supplementary material for: Sparse Gamma Rhythms Arising through Clustering in Adapting Neuronal Networks
Source: PLoS Comput Biol. 2011 Nov 17;7(11):e1002281. doi: 10.1371/journal.pcbi.1002281 (PMC3219625; doi:10.1371/journal.pcbi.1002281)
Supplement: Text S1 — Singular perturbation theory and exact calculation of periodic solution to idealized spiking model with adaptation. (PDF) [file pcbi.1002281.s001.pdf]

Sparse gamma rhythms arising through clustering in adapting neuronal networks: Supplementary information (S1)

Zachary P. Kilpatrick, G. Bard Ermentrout
Department of Mathematics, University of Pittsburgh, Pittsburgh, PA
∗ E-mail: zpkilpat@pitt.edu

Singular perturbation theory and exact calculation of periodic solution to idealized spiking model with adaptation

The following text consists of two companion sections to the main manuscript, *Sparse gamma rhythms arising through clustering in adapting neuronal networks*. The first section details an approximation of the periodic solution of a theta model neuron with spike frequency adaptation. This makes use of the slow timescale of spike frequency adaptation to separate the system into a fast and a slow subsystem, which can be analyzed together using singular perturbation theory. These results are summarized in the main manuscript section entitled **Approximating the periodic solution and cluster number with singular perturbation theory**. The next section exactly calculates the periodic solution of a quadratic integrate-and-fire model neuron with spike frequency adaptation. This is used in the main manuscript section entitled **Phase-resetting curve of an adapting neuron** to compute the phase-resetting curve of the model.

Singular perturbative approximation of periodic solution

In this section, we proceed to compute a singular perturbative approximation to the periodic solution of

$$\begin{aligned} \dot{\theta} &= 1 - \cos\theta + (1 + \cos\theta)(I - \beta z), \\ \dot{z} &= -z/\tau_a + \delta(\pi - \theta), \end{aligned} \tag{1}$$

with period T, such that $\theta(0) = -\pi$ and $\theta(T) = \pi$. With these assumptions, we can solve for

$$z = z_0 e^{-t/\tau_a} = \frac{e^{-t/\tau_a}}{1 - e^{-T/\tau_a}}. \tag{2}$$

Therefore the system (1) reduces to a single nonautonomous equation for the phase variable,

$$\dot{\theta} = 1 - \cos\theta + (1 + \cos\theta)(I - \beta z_0 e^{-\epsilon t}), \tag{3}$$

where we have defined $\epsilon = 1/\tau_a \ll 1$, since we know the adaptation time constant is large, $\tau_a \gg 1$. By ignoring dynamics that occur on the slow timescale $s = \epsilon t$, we can consider a fast subsystem

$$\dot{\theta} = 1 - \cos\theta + (1 + \cos\theta)(I - \beta z_0), \tag{4}$$

which should describe initial dynamics within an initial boundary layer. It is straightforward to solve (4), along with the boundary condition $\theta(0) = -\pi$ to find

$$\theta(t) = 2\tan^{-1}\left[\sqrt{I - \beta z_0}\tan\left(\sqrt{I - \beta z_0}\, t - \frac{\pi}{2}\right)\right] \tag{5}$$

within the initial layer. Once the dynamics of the fast subsystem (5) have settled to their limiting value,

$$\lim_{t\to\infty} 2\tan^{-1}\left[\sqrt{I - \beta z_0}\tan\left(\sqrt{I - \beta z_0}\, t - \frac{\pi}{2}\right)\right] = -\frac{\pi}{2},$$

they will evolve along a manifold determined by the slow subsystem

$$0 = 1 - \cos\theta + (1 + \cos\theta)(I - \beta z_0 \mathrm{e}^{-s}), \tag{6}$$

where $s = \epsilon t$ is a slow time variable. We can solve (6) for the outer layer's dynamics

$$\theta(s) = -\cos^{-1}\left[\frac{I - \beta z_0 \mathrm{e}^{-s} + 1}{\beta z_0 \mathrm{e}^{-s} + 1 - I}\right]. \tag{7}$$

Notice that this solution will vanish when $\beta z_0 \mathrm{e}^{-\epsilon T_{SN}} = I$. This is related to the fact that as the total input to the neuron passes through zero, there is a saddle-node bifurcation in the equilibria structure of the associated fast subsystem [1]. This is a common mechanism for initiating the fast part of a relaxation oscillation [2]. The slow solution will therefore last about

$$T_{SN} = \frac{1}{\epsilon} \ln \frac{\beta z_0}{I}.$$

When the system reaches the vicinity of the saddle-node ($t \approx T_{SN}$), it will begin to evolve according to fast dynamics. Therefore, we must calculate the terminal dynamics of the periodic solution within a boundary layer. To do this, we presume perturbative solutions and fast timescales with arbitrary scaling $\theta = \epsilon^p \theta_1$ and $\tau = \epsilon^q (t - T_{SN})$. Substituting these expressions into (3), we have

$$\epsilon^{p+q} \frac{\mathrm{d}\theta_1}{\mathrm{d}\tau} = \frac{1}{2}\epsilon^{2p}\theta_1^2 + 2\beta z_0 \mathrm{e}^{-\epsilon T_{SN}} \epsilon^{1-q} \tau.$$

Upon setting $p = q = 1/3$, we find the order of all terms is matched. Now, we apply the Riccati transformation $\theta_1 = -2\dot{y}/y$, as well as a change of variables $r = B\tau$, where

$$B = \left(\frac{\beta z_0 \mathrm{e}^{-\epsilon T_{SN}}}{2}\right)^{1/3} = \left(\frac{I}{2}\right)^{1/3}.$$

This yields Airy's equation

$$\frac{\mathrm{d}^2 y}{\mathrm{d}r^2} = ry,$$

which has general solutions

$$y(r) = c_1 \mathrm{Ai}(r) + c_2 \mathrm{Bi}(r),$$

where $\mathrm{Ai}(r)$ and $\mathrm{Bi}(r)$ are the Airy functions of the first and second kind. We specify the solution θ_1 by transforming back, changing variables back to τ, and applying the initial condition $\theta_1(0) = 0$ to find

$$\theta_1(\tau) = 2B \frac{\sqrt{3}\mathrm{Ai}'(-B\tau) + \mathrm{Bi}'(-B\tau)}{\sqrt{3}\mathrm{Ai}(-B\tau) + \mathrm{Bi}(-B\tau)}.$$

We can predict the point where the inner layer solution will diverge to be the minimal τ_b such that $\tau_b > 0$ and

$$\sqrt{3}\mathrm{Ai}(-B\tau_b) = -\mathrm{Bi}(-B\tau_b). \tag{8}$$

The blow up of this inner solution roughly denotes the end of the solution period. Converting back to the time variable t, we find the period will be

$$\begin{aligned} T &= T_{SN} + \frac{\tau_b}{\epsilon^{1/3}} \\ &= \frac{1}{\epsilon} \ln \frac{\beta z_0}{I} + \frac{\tau_b}{\epsilon^{1/3}}. \end{aligned} \tag{9}$$

Substituting (9) into (2) and requiring self-consistency, we can solve for the initial condtion

$$z_0 = 1 + \frac{I}{\beta}\mathrm{e}^{-\epsilon^{2/3}\tau_b}.$$

Therefore, the time it takes to reach the saddle-node is

$$\begin{aligned} T_{SN} &= \frac{1}{\epsilon}\ln\left[\frac{\beta}{I} + \mathrm{e}^{-\epsilon^{2/3}\tau_b}\right] \\ &\approx \frac{1}{\epsilon}\left\{\ln\left[\frac{\beta}{I}+1\right] - \frac{\epsilon^{2/3}\tau_b}{\beta/I+1}\right\}, \end{aligned} \quad (10)$$

when we Taylor expand to first order. Plugging (10) into (9) and rewriting $\tau_a = 1/\epsilon$, we have the approximation for the period of the solution

$$T \approx \tau_a \ln\left[\frac{\beta}{I}+1\right] + \frac{\beta\tau_a^{1/3}\tau_b}{\beta+I},$$

where τ_b is determined by (8).

Note that the outer solution (7) becomes undefined once the saddle-node of the fast subsystem is reached at $t = T_{SN}$. Thus, we must construct the singular solution in a piecewise manner with two regions, where one region is the sum of the initial and outer layers and another region is the terminal layer. Using the timescale t and noting $\epsilon = 1/\tau_a$, we can write

$$\begin{aligned} \theta(t) &= 2\tan^{-1}\left[\sqrt{I-\beta z_0}\tan\left(\sqrt{I-\beta z_0}t - \frac{\pi}{2}\right)\right] + \frac{\pi}{2} \\ &\quad - \cos^{-1}\left[\frac{I-\beta z_0\mathrm{e}^{-t/\tau_a}+1}{\beta z_0\mathrm{e}^{-t/\tau_a}+1-I}\right], \quad t \in (0, T_{SN}), \end{aligned}$$

and

$$\theta(t) = \frac{2B}{\tau_a^{1/3}}\frac{\sqrt{3}\mathrm{Ai}'(B(T_{SN}-t)/\tau_a^{1/3}) + \mathrm{Bi}'(B(T_{SN}-t)/\tau_a^{1/3})}{\sqrt{3}\mathrm{Ai}(B(T_{SN}-t)/\tau_a^{1/3}) + \mathrm{Bi}(B(T_{SN}-t)/\tau_a^{1/3})}, \quad t \in (T_{SN}, T).$$

Exact periodic solution for quadratic integrate-and-fire model with spike frequency adaptation

In this section, we explicitly solve for a periodic solution to

$$\begin{aligned} \dot{x} &= x^2 + I - \beta z, \\ \dot{z} &= -z/\tau_a + \delta(1/x). \end{aligned} \quad (11)$$

To do so, we require the boundary conditions $x(0) = -\infty$ and $x(T) = \infty$. We can immediately solve the equation for the adaptation variable

$$z(t) = \frac{\mathrm{e}^{-t/\tau_a}}{1-\mathrm{e}^{-T/\tau_a}}.$$

Assigning the parameters $\epsilon = 1/\tau_a$ and $\bar{\beta} = \beta/(1-\mathrm{e}^{-\epsilon T})$, we can express the equation for x now as

$$\frac{\mathrm{d}x}{\mathrm{d}t} = x^2 + I - \bar{\beta}\mathrm{e}^{-\epsilon t}. \quad (12)$$

Note, we use ϵ here for comparison with our singular perturbation theory results. Our next step is to employ the transformation $x = -\dot{y}/y$ to convert the Riccati equation (12) to

$$\frac{\mathrm{d}^2 y}{\mathrm{d}t^2} = [\bar{\beta}\mathrm{e}^{-\epsilon t} - I]y, \tag{13}$$

a second order linear equation. Now, by making the change of variables $r = \mathrm{e}^{-\epsilon t/2}$, we can in fact convert (13) to

$$r^2 \frac{\mathrm{d}^2 y}{\mathrm{d}r^2} + r\frac{\mathrm{d}y}{\mathrm{d}r} = \frac{4}{\epsilon^2}\left[\bar{\beta}r^2 - I\right] y.$$

Upon employing a change to imaginary variables $\mu = 2\bar{\beta}ir/\epsilon$ and $\nu = 2\sqrt{I}i/\epsilon$, we find $y(\mu)$ is described by Bessel's equation

$$\mu^2 \frac{\mathrm{d}^2 y}{\mathrm{d}\mu^2} + \mu\frac{\mathrm{d}y}{\mathrm{d}\mu} + \left[\mu^2 - \nu^2\right] y = 0,$$

whose general solutions are given

$$y(\mu) = c_1 J_\nu(\mu) + c_2 Y_\nu(\mu),$$

where $J_\nu(\mu)$ and $Y_\nu(\mu)$ are Bessel functions of the first and second kind, respectively. Changing the constant ν and variable μ back, we find y is given as the sum of Bessel functions with imaginary order and argument

$$y(t) = c_1 J_{2\sqrt{I}i/\epsilon}\left(\frac{2\sqrt{\bar{\beta}}i}{\epsilon}\mathrm{e}^{-\epsilon t/2}\right) + c_2 Y_{2\sqrt{I}i/\epsilon}\left(\frac{2\sqrt{\bar{\beta}}i}{\epsilon}\mathrm{e}^{-\epsilon t/2}\right).$$

We find that, by requiring that the left boundary condition, $x(0) = -\infty \Rightarrow y(0) = 0$, be satisfied, the solution y is restricted to be of the form

$$y(t) = c_1 \mathrm{Im}\left\{ J_{2\sqrt{I}i/\epsilon}\left(\frac{2\sqrt{\bar{\beta}}i}{\epsilon}\mathrm{e}^{-\epsilon t/2}\right)\right\},$$

so that the period T can be specified by the right boundary condition, $x(T) = \infty \Rightarrow y(T) = 0$, so

$$y(T) = \mathrm{Im}\left\{ J_{2\sqrt{I}i/\epsilon}\left(\frac{2i}{\epsilon}\sqrt{\frac{\bar{\beta}}{\mathrm{e}^{\epsilon T} - 1}}\right)\right\} = 0.$$

This fully characterizes the solution, since the remaining constant c_1 is eliminated by the form of $x(t) = -\dot{y}(t)/y(t)$. In addition, since we now have a formula for the periodic solution to the system (11), we can compute the associated adjoint, related to the phase resetting curve.

References

1. Guckenheimer J, Hoffman K, Weckesser W (2000) Numerical computation of canards. Int J Bifurcat Chaos 10: 2669–2687.

2. Mishchenko EF, Rozov NK (1980) Differential Equations with Small Parameters and Relaxation Oscillations. Plenum Press, New York.
